# Rapid detection of invasive *Mycobacterium chimaera* disease via a novel plasma-based next-generation sequencing test

**DOI:** 10.1186/s12879-019-4001-8

**Published:** 2019-05-02

**Authors:** Jim Nomura, Gunter Rieg, Gary Bluestone, Townson Tsai, Andrew Lai, Dawn Terashita, Sivan Bercovici, David K. Hong, Brian P. Lee

**Affiliations:** 1 Southern California Permanente Medical Group, Infectious Disease Department, Los Angeles Medical Center, 1505 N Edgemont St, Los Angeles, CA 90027 USA; 2 South Bay Medical Center, Harbor City, CA USA; 3 Baldwin Park Medical Center, Baldwin Park, CA USA; 4 San Diego Medical Center, San Diego, CA USA; 5 Los Angeles County Department of Public Health, Los Angeles, CA USA; 6 Karius, Inc, 975 Island Drive, Suite 101, Redwood City, CA 94065 USA

**Keywords:** *Mycobacterium chimaera* infection, Heater-cooler devices, Cell-free DNA, Next-generation sequencing

## Abstract

**Background:**

There is an ongoing outbreak of *Mycobacterium chimaera* infections among patients exposed to contaminated heater-cooler devices used during cardiac surgery. Recognition of *M. chimaera* infection is hampered by its long latency and non-specific symptoms. Standard diagnostic methods using acid-fast bacilli (AFB) culture often require invasive sampling, have low sensitivity, and can take weeks to result. We describe the performance of a plasma-based next-generation sequencing test (plasma NGS) for the diagnosis of *M. chimaera* infection.

**Methods:**

We conducted a retrospective study of 10 patients with a history of cardiac surgery who developed invasive *M. chimaera* infection and underwent testing by plasma NGS between February 2017 and April 2018.

**Results:**

Plasma NGS detected *M. chimaera* in 9 of 10 patients (90%) with invasive disease in a median of 4 days from specimen collection, including all 8 patients with disseminated infection. In 7 of these 9 cases (78%), plasma NGS was the first test to provide microbiologic confirmation of *M. chimaera* infection. In contrast, AFB cultures required a median of 20 days to turn positive, and the median time for confirmation of *M. chimaera* was 41 days. Of 24 AFB blood cultures obtained in this cohort, only 4 (17%) were positive. Invasive procedures were performed in 90% of cases, and in 5 patients (50%), mycobacterial growth was achieved only by culture of these deep sites.

**Conclusions:**

Plasma NGS can accurately detect *M. chimaera* noninvasively and significantly faster than AFB culture, making it a promising new diagnostic tool.

**Electronic supplementary material:**

The online version of this article (10.1186/s12879-019-4001-8) contains supplementary material, which is available to authorized users.

## Background

*Mycobacterium chimaera,* a nontuberculous mycobacterium belonging to the *Mycobacterium avium* complex (MAC), is an opportunistic human pathogen that is ubiquitous in the environment, particularly in water sources [1]. *M. chimaera* contamination of the LivaNova 3 T (LivaNova, London, UK) heater-cooler device (HCD) used for thermoregulation during cardiothoracic surgery has been linked to an ongoing global outbreak of serious infections, including in Europe [2–7], North America [7–10], Australia and New Zealand [11, 12]. During HCD operation, the organism has been demonstrated to aerosolize via the exhaust of contaminated devices, resulting in airborne inoculation of the surgical site [13]. Whole genome sequencing of *M. chimaera* isolates from infected patients and HCD water samples worldwide has demonstrated sequence similarity, suggesting point source contamination at the time of device manufacturing [7, 14, 15]. The potential for widespread exposure to infection is significant; in the US alone, LivaNova 3 T HCDs have been in use since 2006, comprise over 60% of the HCD market, and are utilized for over 250,000 cardiac surgeries each year [16, 17]. While infection can be localized to the sternal wound, disseminated disease involving the liver, spleen, bone marrow, kidney, bones, joints or eyes has been typical among cardiac surgery patients with implanted prosthetic material [4, 8, 16]. Reported mortality has been as high as 50% [16].

There are several challenges inherent in the diagnosis of *M. chimaera* infection. Clinical symptoms, including fatigue, fever, sweats, cough, dyspnea and weight loss, are non-specific and indolent, manifesting as long as 6 years after surgery [16]. Standard confirmatory testing utilizing acid-fast bacilli (AFB) culture often requires invasive sampling, has limited sensitivity, and can take up to 8 weeks for mycobacterial growth. While some clinical laboratories can identify MAC isolates from AFB culture, the specialized molecular methodologies required for differentiation of *M. chimaera* are typically available only at reference or research laboratories, further delaying confirmation of the causative pathogen [2–4].

Given these challenges, there is an urgent need for innovative diagnostic approaches. Advances in next-generation sequencing (NGS) and bioinformatics have been utilized for the direct detection of pathogen nucleic acid and hold great promise, but invasive procedures are generally necessary to obtain appropriate clinical specimens for testing [18]. The application of NGS to a blood sample is one way to overcome this hurdle and has been used successfully in other areas of medicine for noninvasive diagnosis [19–23]. This approach takes advantage of the cell-free DNA (cfDNA) present in plasma that can be derived from potentially any source within the body, including pathogens at deep sites of infection [20, 24–26]. In this report, we describe the application of a plasma-based NGS test for the diagnosis of invasive *M. chimaera* infection.

## Methods

### Patient identification and specimens

This was a retrospective study of patients with a history of cardiac surgery performed at a Southern California hospital with known *M. chimaera* exposure risk who developed invasive *M. chimaera* infection and who had testing of cell-free plasma by NGS (plasma NGS) between February 2017 and April 2018. Cases were defined as patients with *M. chimaera* identified from AFB culture. AFB cultures of blood, urine, sputum, and biopsy samples and identification of isolates to the level of MAC were performed at the hospital’s regional laboratory. Speciation by partial 16S rRNA gene sequencing was performed at the University of Texas at Tyler.

Plasma NGS testing was performed at Karius, Inc. (Redwood City, CA), a reference laboratory with Clinical Laboratory Improvement Amendments certification and College of American Pathologists accreditation. A broad plasma NGS was validated to detect over 1000 microorganisms, including bacteria (including *M. chimaera*), DNA viruses, and eukaryotic pathogens including yeasts, mold, and protozoa, and was used for patients 1–6. In May 2017, an *M. chimaera* plasma NGS test was validated and was used for patients 7–10.

For plasma NGS, whole blood (minimum of 4 mL) was collected in BD Vacutainer™ K_2_EDTA Blood Collection tubes (Becton, Dickinson and Company, New Jersey, US) or in plasma preparation tubes (PPT) via a peripheral blood draw. Samples in K_2_EDTA tubes were centrifuged at 1600 rcf for 10 min within 72 h of collection, and separated plasma was aliquoted into a sterile polypropylene tube. Samples in PPTs were centrifuged at 1100 rcf for 10 min within 6 h of collection to separate the plasma. Processed specimens were then shipped at ambient temperature to Karius, Inc.

### Sequencing

The plasma sample was centrifuged at 16,000 rcf for 10 min at room temperature and spiked with a known concentration of synthetic DNA molecules for quality control purposes. Cell-free DNA was extracted from 0.5 mL plasma using a magnetic bead-based method (Omega Biotek, Norcross, GA), typically yielding 0.1 ng to 10 ng to be used for sequencing. DNA libraries for sequencing were constructed using a modified Ovation® Ultralow System V2 library preparation kit (NuGEN, San Carlos, CA). Negative controls (buffer only instead of plasma) and positive controls (healthy human plasma spiked with a known mixture of microbial DNA fragments) were processed alongside patient samples in every batch. Samples were multiplexed and sequenced on the Illumina NextSeq® 500 (Illumina, Inc., San Diego, CA).

### Bioinformatics analysis pipeline

Primary sequencing output files were processed using bcl2fastq (v2.17.1.14) to generate the demultiplexed sequencing reads files. Reads were filtered based on sequencing quality and trimmed based on partial or full adapter sequence. The bowtie2 (version 2.2.4) method was used to align the high-quality reads against human and synthetic-molecules references. Sequencing reads that aligned to the human or synthetic molecule references were removed from further analysis. Remaining reads were aligned against Karius’ proprietary microorganism reference database using NCBI-blast (version 2.2.30). Only taxa present at statistically significant levels above background were reported. The entire process from DNA extraction through analysis is typically completed within 28 h. For full analysis details and evaluation of the bioinformatics analysis pipeline for the detection of *M. chimaera*, see Additional file 1: Supplementary Methods Appendix.

## Results

### Illustrative case (Fig. 1 & Table 1, patient 1)

A 54-year old male, who had undergone aortic valve and root replacement 17 months prior, was admitted with a 2-month history of fatigue, night sweats, and weight loss. Laboratory studies revealed pancytopenia, but transesophageal echocardiogram (TEE) showed no vegetations. AFB blood cultures and plasma NGS were drawn upon admission, and the patient was empirically treated with azithromycin, rifabutin, ethambutol, and amikacin.

**Fig. 1:**
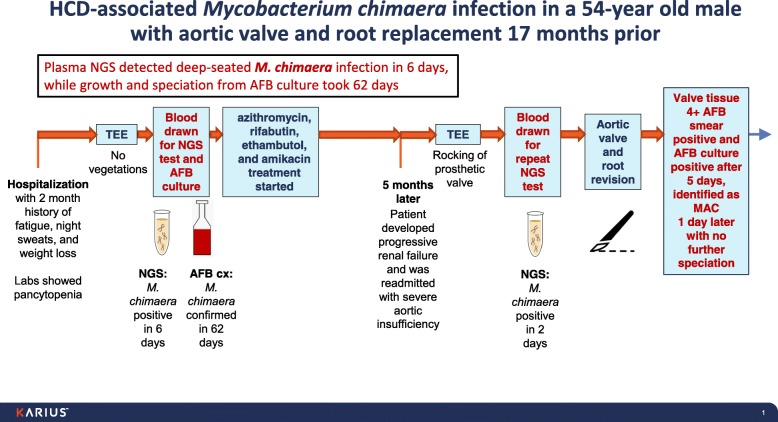
Timeline of the clinical course for patient 1 (illustrative case). Plasma NGS detected *M. chimaera* infection in 6 days compared to 62 days for growth and speciation from AFB blood culture

**Table 1:** Summary of patients with invasive *M. chimaera* disease

Patient	Preceding cardiac surgery (months prior to presentation)	Presenting symptoms (duration in months)	Clinical scenario (disease type)	Specimen source	Days to positive AFB culture	Days to identify MAC from AFB culture	Days to speciate *M. chimaera* from AFB culture	Days to detect *M. chimaera* by plasma NGS^a^	Additional organisms detected by plasma NGS	Days on MAC therapy at time of plasma NGS (regimen)	Patient outcome
1 (illus-trative case)	AVR, ARR (17)	Fatigue, weight loss, night sweats (4)	Pancytopenia, renal failure, severe aortic insufficiency and rocking of prosthetic valve (disseminated)	Blood (1 of 3 positive)	43	47	62	6^c^	None	0 (azithromycin, ethambutol, rifabutin, amikacin)	Alive (s/p redo surgery)
				Valve	5	6	Not done				
2	AVR, ARR (14)	Fatigue, weight loss, cough, fever, night sweats (4)	Pancytopenia, granulomatous hepatitis and nephritis (disseminated)	Blood (4)	Negative	N/A	N/A	3^c^	None	12 (azithromycin, ethambutol, rifampin)	Expired
				Bone marrow	Negative	N/A	N/A				
				Liver	14	17	43				
				Urine	18	20	41				
3	AVR, MVR (22)	Nausea and chills (3)	Pancytopenia, large MV vegetation, granulomatous hepatitis and bone marrow granulomas (disseminated)	Blood (2)	Negative	N/A	N/A	5^c^	EBV	0 (azithromycin, ethambutol, rifabutin, amikacin)	Expired
				Bone marrow	20	22	Not done				
				Valve	6	8	23				
4	AVR (17)	Lower back pain (2)	T11-T12 diskitis and vertebral osteomyelitis (disseminated)	Disk	20	23	39	2	EBV	22 (azithromycin, ethambutol, rifabutin)	Expired
5	AVR, ARR (21)	Fatigue, fever, night sweats (3)	Fluid collection surrounding aorta (localized)	Blood (3)	Negative	N/A	N/A	Negative	None	0 (azithromycin, ethambutol, rifampin, amikacin, moxifloxacin)	Alive (s/p redo surgery)
				Graft/valve	24	27	41				
6	AVR (14)	Weakness, weight loss, fever, night sweats (3)	Thrombocyto-penia, granulomatous hepatitis (disseminated)	Liver	26	35	92	6	*Actinomyces oris*, *Prevotella corporis*, *Prevotella buccalis*, *Gardnerella vaginalis*, *Acinetobacter baumannii*	103 (clarithromycin, ethambutol, rifabutin)	Expired
7	AVR, ARR (27)	Dyspnea, lower extremity edema, weight loss (5)	Anemia, thrombocyto-penia, cholestatic hepatitis, aortic root abscess (disseminated)	Blood (1 of 5 positive)	28	28	Not done	2^c^	N/A^b^	0 (azithromycin, ethambutol, rifampin, moxifloxacin)	Expired
				Sputum	17	20	38				
				Bone marrow	25	26	34				
8	AVR (25)	Weakness, cough, fever (0.75)	Pancytopenia, cholestatic hepatitis (disseminated)	Blood (1)	Negative	N/A	N/A	3^c^	N/A^b^	3 (azithromycin, ethambutol, rifampin, moxifloxacin, amikacin)	Alive (s/p redo surgery)
				Sputum	23	Not done	42				
				Bone marrow	27	29	Not done				
				Liver	24	27	Not done				
				Valve	6	10	34				
9	AVR, ARR (12)	Chest pain, weight loss, fatigue (9)	Anemia, mediastinal mass (localized)	Blood (4)	Negative	N/A	N/A	4^c^	N/A^b^	0 (azithromycin, ethambutol, rifampin, moxifloxacin)	Alive (s/p redo surgery)
				Urine (2)	Negative	N/A	N/A				
				Mediastinal lymph node	15	24	43				
10	AVR, ARR (21)	Weakness, fatigue, weight loss, fever (2)	Pancytopenia, bone marrow granulomas (disseminated)	Blood (2 of 2 positive)	14	17	46	4^c^	N/A^b^	0 (azithromycin, ethambutol, rifabutin)	Expired
					15	18	Not done				

*AVR* = aortic valve replacement, *ARR* = aortic root replacement, *MVR* = mitral valve replacement, *AFB* = acid-fast bacilli, *MAC* = *Mycobacterium avium* complex, *NGS* = next-generation sequencing

^a^Includes only the first positive plasma NGS

^b^Dedicated *M. chimaera* plasma NGS

^c^NGS provided the first microbiological confirmation of *M. chimaera* infection. For patients 4 and 6, the plasma NGS was not performed until approximately 6 weeks and 4.5 months after AFB cultures had been collected

Plasma NGS detected *M. chimaera* 6 days later. The patient improved clinically and was discharged on oral therapy. One of three AFB blood cultures turned positive after 43 days. The isolate was identified as MAC 4 days later but speciation to *M. chimaera* required an additional 15 days, a total of 62 days from specimen collection.

Five months later, the patient developed progressive renal failure and was readmitted with severe aortic insufficiency and rocking of the prosthetic valve seen on TEE. Repeat plasma NGS was sent, and in 2 days, *M. chimaera* was again detected. The patient underwent aortic valve and root revision. Valve tissue was 4+ AFB smear positive and AFB culture was positive after 5 days, with identification as MAC one day later and no further speciation.

### Case series overview

From February 2017 to April 2018, ten patients with invasive *M. chimaera* disease, all confirmed by standard AFB culture, who had undergone testing by plasma NGS were identified (Table 1). The median age of this cohort was 65.5 years (range: 51–79 years), and 80% were male. All had undergone prior aortic valve replacement and 60% had also had aortic root replacement. Initial clinical symptoms developed a median of 19 months (range: 12–27 months) after surgery and included weight loss (50%), fatigue (40%), fever (40%), night sweats (40%), and cough (20%). The median duration of symptoms prior to evaluation for mycobacterial infection was 3 months (range: 0.75–9 months). The majority of patients (80%) had evidence of disseminated disease involving the liver, kidney, bone marrow and/or vertebral disk and bone, whereas 2 patients had deep infection localized to the original surgical area (aorta and mediastinum). Despite antimycobacterial treatment, mortality was 60%.

One or more invasive procedures, such as biopsy of liver, bone marrow, disk, or lymph node or cardiac valve resection, were performed in 90% of cases in order to obtain specimens for AFB culture. In 5 cases (50%), growth of mycobacteria was achieved only by culture of these deep sites. In the remaining patients, blood, sputum, and/or urine for AFB culture was diagnostic. Of note, a total of 24 AFB blood cultures were obtained in this cohort, and only 4 (17%) were positive.

**Fig. 2:**
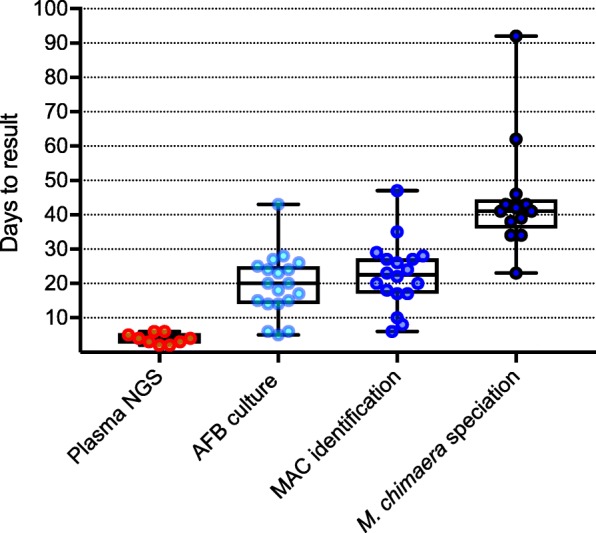
Number of days to positive test result (from sample collection): plasma NGS (9 samples): median of 4 days; AFB culture (19 samples): median of 20 days; MAC identification (18 samples): median of 22.5 days; *M. chimaera* speciation (13 samples): median of 41 days

Overall, AFB cultures from any site were positive in a median of 20 days (range: 5–43 days); identification of MAC took a median of 3 additional days (range: 0–9 days), and further speciation to *M. chimaera* required a median of 19 additional days (range: 8–57 days). From specimen collection for AFB culture, the median total time required for confirmation of *M. chimaera* was 41 days (range: 23–92 days) (Fig. 2).

### Performance of plasma NGS

Plasma NGS detected *M. chimaera* in 90% of these patients with invasive disease in a median of 4 days from specimen collection (range: 2–6 days), including all 8 patients (100%) with disseminated disease and 1 of 2 patients (50%) with localized disease. In 7 of these 9 cases (78%), plasma NGS was the first test to provide microbiologic confirmation of *M. chimaera* infection (Table 1); in the other 2 cases, the plasma NGS was not performed until approximately 6 weeks and 4.5 months after AFB cultures had been collected. Five of the 9 positive tests (56%) were obtained prior to initiation of antimycobacterial therapy; the remaining 4 (44%) were collected while on treatment for a median of 17 days (range: 3–103 days).

Of the 6 positive broad plasma NGS tests performed in this cohort, three (50%) detected only *M. chimaera* while two (in patients 3 and 4) detected EBV and one (in patient 6) detected 5 microorganisms (*Actinomyces oris*, *Prevotella corporis*, *Prevotella buccalis*, *Gardnerella vaginalis*, and *Acinetobacter baumannii*) in addition to *M. chimaera*. Of note, EBV PCR of the blood drawn from patient 3 was positive at 600 copies/mL.

## Discussion

To our knowledge, this is the first report describing the successful application of a plasma-based NGS test for the direct detection of *M. chimaera*, providing microbiologic confirmation of this fastidious organism noninvasively and over 1 month faster than standard AFB culture. Cases included in this series were typical of other patients with invasive *M. chimaera* reported in the literature with regard to the type of exposure, presence of prosthetic material, long incubation period, nonspecific presenting symptoms, disease manifestations, and mortality [2–12]. As in prior reports, confirmation of the diagnosis was typically delayed when reliant solely on AFB culture, and invasive biopsies were often necessary to grow the pathogen from deep-seated infections. The median time to AFB growth was 20 days with a median of 3 additional days to identify isolates as MAC, and a median of 19 additional days to speciate to *M. chimaera*. The median overall time from AFB culture collection to confirmation of *M. chimaera* was 41 days. In contrast, plasma NGS detected this pathogen in a median of 4 days, and in 78% of cases, this provided the first microbiologic confirmation. In four cases, plasma NGS detected *M. chimaera* weeks to months after initiation of antimycobacterial therapy, demonstrating that the pathogen cfDNA signal may persist even in the face of antibiotic pretreatment.

Although early diagnosis of *M. chimaera* infection has the potential to improve patient outcomes, this benefit was difficult to demonstrate in this small study. The mortality rate for our overall cohort was 60%, and this rate was similar even in the subset for whom plasma NGS was the first to provide microbiologic confirmation (7 patients) as well as the subset in whom *M. chimaera* was detected by plasma NGS prior to the start of antimycobacterial therapy (5 patients). Among the 4 patients who survived their infection, all underwent redo cardiac surgery, suggesting that removal of the infected prosthesis may be crucial. In 3 of these patients, plasma NGS was the first to provide microbiologic confirmation of *M. chimaera* infection. Thus, timely diagnosis could potentially improve mortality by informing early surgical intervention.

The application of NGS for the detection of cfDNA in plasma has been successfully employed for noninvasive diagnosis in other areas of medicine. Prenatal detection of fetal chromosomal defects can be accomplished by the sequencing of fetal cfDNA in maternal circulation, obviating the need for amniocentesis [19]. Organ rejection in solid organ transplant recipients has been monitored by sequencing of tumor-derived cfDNA in the recipient’s blood [21, 22]. In some cases, the diagnosis of cancer can be made without biopsy via sequencing of tumor-derived cfDNA in a blood sample [23].

In contrast to these applications, which focus on human cfDNA, the plasma NGS test described in this report targets microbial cfDNA in the plasma for pathogen detection. This approach has previously been used to rapidly detect another fastidious bacterium, *Capnocytophaga canimorsus*, in an asplenic patient presenting with culture-negative sepsis [24]. In addition, the same plasma NGS technology was performed on nine patients with proven invasive fungal infection and was able to detect the same fungus identified from biopsy tissue in 7 cases (78%) [25]. Similar to the current study, this series demonstrated that pathogen cfDNA from deep-seated infections caused by difficult-to-culture organisms, including molds like *Aspergillus*, *Rhizomucor*, and *Scedosporium* species, can be detected by NGS applied to a cell-free plasma sample, potentially providing a more rapid diagnosis and obviating the need for invasive biopsies.

Despite the promise of NGS technology, some limitations remain [18]. The kinetics of how pathogen cfDNA enters the plasma space is poorly understood and may differ by pathogen and by site and severity of infection. In this small cohort, plasma NGS was positive in 100% of patients with disseminated infection but in only one of two patients with localized infection, suggesting that the abundance of pathogen cfDNA available in the plasma space may be lower with milder or limited disease. The sole case not detected by plasma NGS (patient 5) was found to have a focal fluid collection surrounding his aorta at the previous surgical site and had no other evidence of disease extension, including 3 negative AFB blood cultures. In addition, broad NGS tests may detect more than one microorganism, and discerning which represent co-pathogens, commensals, or contaminants can prove challenging. The detection of EBV in two of our patients (patients 3 and 4) may have represented viral reactivation; in the former, EBV PCR of the blood corroborated the plasma NGS result. In patient 6, we suspect that the mixed mucosal and environmental organisms detected were due to low-level mucosal translocation or sample contamination. Despite this concern, a broad NGS test could be useful in many clinical settings for the detection of unsuspected pathogens or co-pathogens. However, this breadth may not be necessary when a specific pathogen is suspected, and for this reason, a dedicated *M. chimaera* plasma NGS was validated and made available. Finally, this particular study was limited by its small sample size. To truly define test performance characteristics such as sensitivity, specificity, and positive and negative predictive values, larger studies are needed [27]. Nevertheless, this case series serves as a pilot study that demonstrates the potential of this novel technology.

## Conclusions

Considering the widespread exposure to contaminated HCDs, the challenges with recognition and diagnosis of *M. chimaera* infection, and the high case fatality rate, there is an urgent need for sensitive and noninvasive tests to detect this pathogen in a clinically actionable time frame. This plasma NGS test rapidly and noninvasively detected *M. chimaera* in 90% of patients with deep-seated infection and is a promising new diagnostic tool for clinicians.

## Additional file

Additional file 1: Supplementary Methods Appendix. This document describes the analytical components of the plasma next-generation sequencing methods in greater detail. (DOCX 603 kb)

## Abbreviations

AFB: Acid-fast bacilli
cfDNA: cell-free DNA
EBV: Epstein-Barr virus
HCD: Heater-cooler device
K2EDTA tube: K2- Ethylenediaminetetraacetic acid tube
MAC: *Mycobacterium avium* complex
NGS: Next-generation sequencing
PCR: Polymerase chain reaction
PPT: Plasma preparation tube
TEE: transesophageal echocardiogram
