# Supplementary material for: Rapid detection of invasive Mycobacterium chimaera disease via a novel plasma-based next-generation sequencing test
Source: BMC Infect Dis. 2019 May 2;19:371. doi: 10.1186/s12879-019-4001-8 (PMC6498503; doi:10.1186/s12879-019-4001-8)
Supplement: Supplementary file 1 — Supplementary Methods Appendix. This document describes the analytical components of the plasma next-generation sequencing methods in greater detail. (DOCX 603 kb) [file 12879_2019_4001_MOESM1_ESM.docx]

**SUPPLEMENTARY METHODS APPENDIX**

***Bioinformatics analysis pipeline and sequence alignment***

Negative controls (buffer only instead of plasma) and positive controls (healthy human plasma spiked with a known mixture of microbial DNA fragments) were processed alongside patient samples in every batch. Primary sequencing output files were processed using bcl2fastq (v2.17.1.14) to generate the demultiplexed sequencing reads files. Reads were filtered based on sequencing quality and trimmed based on partial or full adapter sequence. The bowtie2 (version 2.2.4) method was used to align the remaining reads against Karius' human and synthetic-molecules references.

Sequencing reads that aligned to the human reference sequence, synthetic-molecule references, and mitochondrial DNA or plasmids were removed from further analysis. Alignments with high percent identity over a substantial portion of the read were retained. Based on the resulting alignments, duplicate sequencing reads were marked and removed. The remaining reads were aligned against the Karius’ proprietary microorganism reference sequence database using National Center for Biotechnology Information (NCBI)-blast (version 2.2.30).

The probability of the observed reads originating from the various taxa represented in the sequence database was computed, accounting for the divergence between the sampled analyte and the reference genomes. An Expectation Maximization algorithm was applied to infer the maximum likelihood estimate for the relative abundance of each taxon. Subsequently, an estimated number of unique reads (accounting for duplicates) was computed by multiplying the relative abundance of a taxon by the total number of unique reads aligning against that taxon. From these abundances, the estimated number of reads arising from each taxon was aggregated up the taxonomic tree. To account for microorganism cell-free DNA that may have been introduced during sample processing (including from the reagents), the relative abundances of taxa in control samples accompanying each sequencing batch were estimated. Results were aggregated from these control samples to derive per-taxon background abundance estimates. To determine whether the levels observed in the samples exceeded those expected to originate from the environment alone, a Poisson model parameterized by the estimated background abundances was applied. Only taxa that rejected this null hypothesis at high significance levels (p < 10^-35^ for broad plasma NGS and p < 10^-10^ for *M. chimaera* plasma NGS) were reported and included in downstream analyses. Final calls were made after additional filtering was applied, accounting for read location uniformity, read percent identity, and cross-reactivity originating from higher abundance calls.

***Microorganism reference sequence database***

Human reference genomes were retrieved from NCBI, including the human assembly GRCh38.p7, as well as additional human references. Sequence reference files for reportable microorganisms, including bacteria, fungi, viruses, protozoa, nematoda, platyhelminthes, and archaea, were downloaded from the NCBI's assembly database (https://www.ncbi.nlm.nih.gov/refseq/, July 2016). The corresponding assemblies were filtered based on their quality annotations and only reference sequences with indicators of high quality assembly (e.g. completeness, N50 score, etc.) were included.

The final set of references comprised more than 22,000 unique assemblies that passed the quality criteria and comprise Karius’ proprietary microorganism reference sequence database. The Taxonomy Database from NCBI, together with the reference database, served as the data foundation for the applied metagenomic abundance estimation algorithm. A subset of these taxa, including over 1,000 clinically significant microorganisms, was used as the clinical reportable range. A full list can be found at <https://www.kariusdx.com/pathogenlist>.

The sequence database is continuously curated to minimize human cross-reactivity as well as cross-reactivity between pathogens and is screened to mitigate contamination with sequences from humans or other organisms.

***Assessment of genetic cross-reactivity between Mycobacterium chimaera and other mycobacteria in reference sequence database***

The potential for genetic sequence cross-reactivity between *M. chimaera* and other mycobacteria, particularly closely related species within MAC, was evaluated using genomic distance mapping analysis. Pairwise genomic distances were calculated among all mycobacterial sequence assemblies in the sequence database and available *M. chimaera* assemblies (references downloaded from NCBI, July 2016). Distances between genome reference pairs were calculated with a variant of the MinHash method,^1^ using k-mers of 21 base pairs in length. None of the *M. chimaera* assemblies contained in the sequence database were deemed at high risk for cross-reactivity with other *Mycobacterium* species (Supplemental Figure). The most closely related species were *M. yongonense* and *M. intracellulare*, two other members of MAC, but their genetic distance was far enough that cross-reactivity was not anticipated.

**
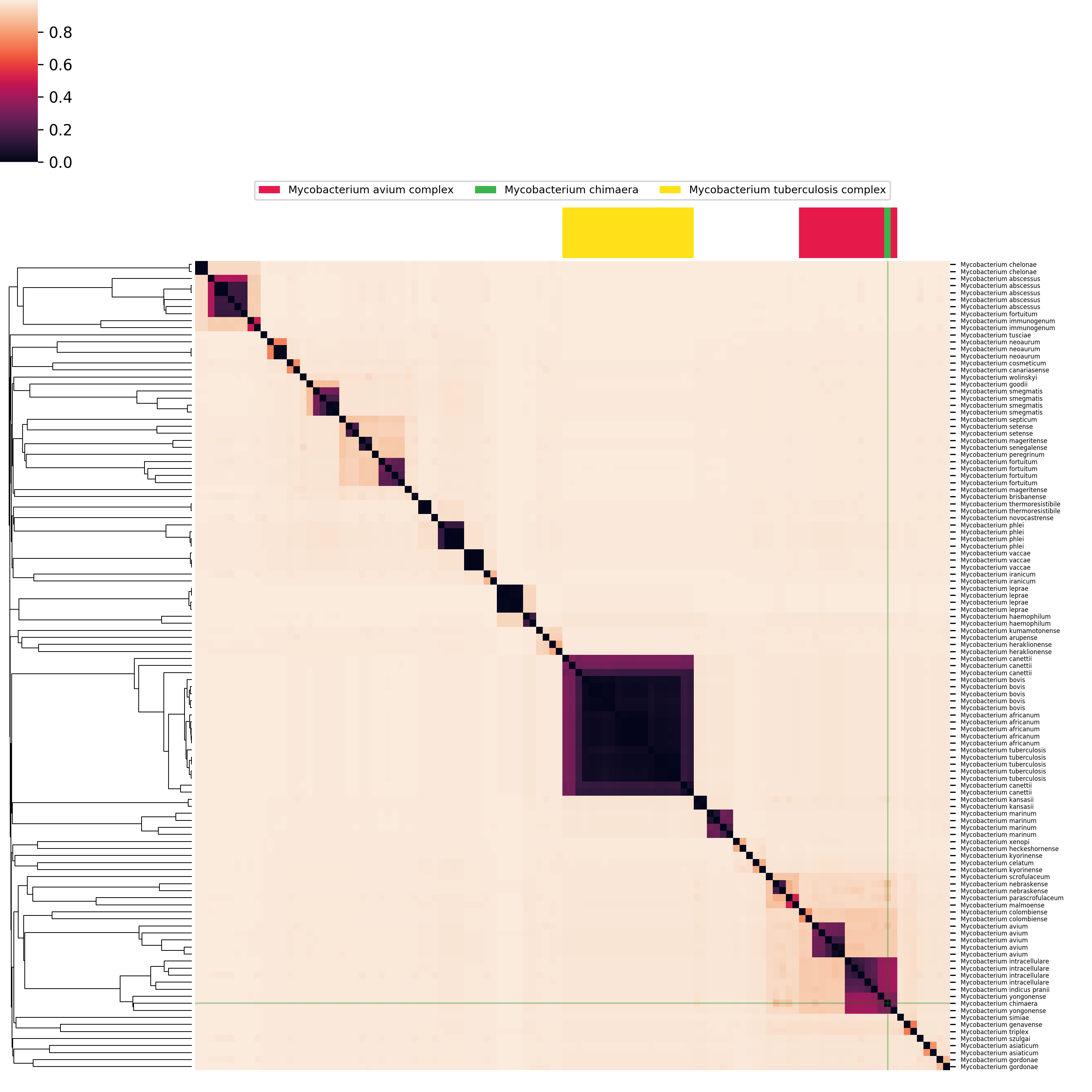
Supplementary Figure:** Similarity-based genome clustering among 115 assemblies, representing 53 *Mycobacterium*species, including *M. chimaera*. For species with more than five assemblies, five were picked at random. Darker colors indicate a greater degree of sequence similarity. Colored bars at the top of the plot correspond to the analyzed species; *M. chimaera* (green) clusters with the other species of the *M. avium complex* (red), with *M. yongonense* as nearest neighbor.

***Assessment of the Bioinformatic Pipeline for detection of M. chimaera***

To assess the performance of the bioinformatic pipeline for identification of *M. chimaera*, computer simulations were conducted. Specificity of the pipeline to detect *M. chimaera* was determined by creating contrived sequence read sets using genetic assemblies from 95 randomly selected *Mycobacterium* species. These read sets were analyzed in a blinded fashion with the pipeline, and there were no false-positive calls of *M. chimaera*. In addition, sensitivity of the pipeline in identifying *M. chimaera* was assessed by downloading full sequence datasets for 48 isolates of *M. chimaera* associated with the current outbreak in Australia and New Zealand (43 from HCDs and 5 from patient samples) from NCBI.^2^ Contrived read sets of 10,000 and 100 reads were created and were analyzed in a blinded analysis with the pipeline. *M. chimaera* was detected at the species level in 48/48 samples at 10,000 reads and in 47/48 samples at 100 reads. In one sample, *M. chimaera* was not detected at 100 reads, however the BAP made the call at the MAC level. This same sample had a clear signal for *M. chimaera* at 10,000 reads, suggesting that the failure to identify *M. chimaera* (and the resulting collapsed identification to MAC) was likely the result of insufficient sequencing depth. No additional organisms were detected in either analysis.

**SUPPLEMENTARY APPENDIX REFERENCES:**

1. Broder, Andrei Z. "On the resemblance and containment of documents." In Compression and Complexity of Sequences 1997. Proceedings, pp. 21-29. IEEE, 1997.
2. Williamson D, Howden B, Stinear T. *Mycobacterium chimaera* spread from heating and cooling units in heart surgery. N Engl J Med 2017;37:600-2. <http://dx.doi.org/10.1056/NEJMc1612023>
